# Metronidazole enhances killing of *Porphyromonas gingivalis* by human PMNs

**DOI:** 10.3389/froh.2022.933997

**Published:** 2022-08-29

**Authors:** Mihaela Anca Serbanescu, Morvarid Oveisi, Chunxiang Sun, Noah Fine, Anne Bosy, Michael Glogauer

**Affiliations:** ^1^Faculty of Dentistry, University of Toronto, Toronto, ON, Canada; ^2^OraVital Inc., Toronto, ON, Canada; ^3^Department of Dental Oncology and Maxillofacial Prosthetics, Princess Margaret Cancer Centre, Toronto, ON, Canada

**Keywords:** metronidazole, periodontitis, Porphyromonas gingivalis, anti-infective agents, neutrophils (PMNs)

## Abstract

**Background and objectives:**

Periodontitis affects the supporting structures of the teeth as a result of the interactions between the subgingival biofilm and the host immune system. Periodontal therapy in severe forms of periodontitis often utilizes antimicrobial agents with some potential to improve host defense responses. In the present study, we investigated the *in vitro* effect of metronidazole (MTZ) at concentrations achievable in the periodontal pocket on PMN activation and PMN mediated killing of *Porphyromonas gingivalis*.

**Materials and methods:**

Flow cytometry based assays were used to measure the impact of MTZ on PMN degranulation, neutrophil extracellular trap (NET) formation and myeloperoxidase (MPO) release and phagocytosis in response to the keystone oral pathogen *P. gingivalis*. Functional assays for PMN mediated killing of P. *gingivalis* and reactive oxygen species (ROS) production in PMN were also carried out.

**Results:**

We demonstrate that PMNs pretreated with MTZ (2 μg/ml or 50 μg/ml) displayed enhanced killing of *P. gingivalis* compared to untreated PMNs. At concentrations achieved physiologically in the periodontal pocket, MTZ induced PMN surface expression of two activation markers (CD66 and CD63). MTZ did not alter *P. gingivalis*-induced NETosis, but suppressed *P. gingivalis*-induced ROS production and phagocytosis.

**Conclusion:**

MTZ displays a positive interaction with PMNs to potentiate PMN mediated killing of *P. gingivalis* and may therefore contribute to its beneficial effects in the treatment of periodontitis initiated by *P. gingivalis* infections including those refractory to conventional treatment.

## Introduction

Periodontitis is a multifactorial polymicrobial infection characterized by inflammatory destruction of tooth-supporting tissues, which results in periodontal pocket formation, alveolar bone resorption, and eventual tooth loss [1]. The host response to subgingival bacteria plays a critical role in periodontal pathogenesis [2]. Polymorphonuclear neutrophils (PMN), are the overwhelming majority (≥ 95%) of leukocytes recruited to the gingival crevice in response to the tooth associated biofilm [3]. During the pathogenesis of periodontal disease, the continuous challenge to the host's immune response and resident cells by periodontal pathogens and their virulence factors results in an enhanced influx of PMNs that participate in inflammation mediated tissue damage and bone resorption [4]. PMNs are continually recruited to the gingival crevice where they form a “defensive barrier” against tooth-associated biofilms to prevent bacterial invasion into the underlying tissues [5].

PMNs are highly reactive cells that use multiple mechanisms to eliminate target pathogens. These include engulfing microbes by phagocytosis [2, 6, 7], reactive oxygen species (ROS) mediated killing, and secretion of toxic granule contents such as myeloperoxidase (MPO) [8]. In addition to this antimicrobial arsenal, highly activated PMNs can release neutrophil extracellular traps (NETs), composed of a decondensed DNA to which histones, proteins (lactoferrin and cathepsins) and enzymes (MPO, elastase)—released from PMN granules are bound [9].

*Porphyromonas gingivalis* is regarded as a key etiological agent involved in the initiation and progression of periodontitis and contributes to the majority of persistent forms of periodontitis [6]. In the course of evolution *P. gingivalis* has developed a number of virulence factors that allow them to avoid and modulate PMN response of the host including impaired recruitment and chemotaxis, resistance to granule-derived antimicrobial agents and oxidative burst, inhibition of phagocytic killing, and delay of PMN netosis [10]. By subverting the immune response of the host, *P. gingivalis* can persist at periodontal sites despite pro-inflammatory recruitment of PMNs, resulting in a chronic dysbiotic state [11]. Furthermore, *P. gingivalis* supports growth of periodontal biofilms through collaboration with other periodontal pathogens [12]. The presence of *P. gingivalis* is closely linked to unresolved periodontal lesions and progressive bone loss [13], and patients with persistence of *P. gingivalis* and other keystone periodontal pathogens do not respond favorably to mechanical debridement, the gold standard in the treatment of periodontal disease [14].

Metronidazole (MTZ), a nitroimidazole antibiotic, is effective against periodontal anaerobic pathogens including *P. gingivalis* [14]. Clinically, MTZ is effective for treating gingivitis, periodontal disease, as a prophylaxis in dental surgery [15–17] and for controlling halitosis in individuals with mild clinical signs of periodontal disease [18]. MTZ was shown to significantly increase attachment gain and reduced probing depth in the deepest probing sites relative to mechanical debridement [15–17]. The effect of MTZ against anaerobic periodontal pathogens for treatment of patients with periodontitis has been described to eliminate *P. gingivalis* from the periodontal pockets [19–21]. Being a prodrug, MTZ is inactive until the nitro group is reduced under low oxygen tension such as periodontal environmental and periodontal tissue. Once MTZ is taken up by periodontal pathogens such as *P. gingivalis* it binds non-specifically to bacterial DNA, leading to DNA breakage, impairment of proper DNA function and destruction of the organism [22]. Some preliminary work suggests that MTZ also has immunomodulatory functions that could contribute to its clinical efficacy [20, 21, 23]. Studies conducted with other antibiotics showed that azithromycin (AZM), amoxicillin (AMX), clarithromycin (CLR), clindamycin (CLD) can also modulate PMN activity with the most significant differences in killing periodontal pathogens observed with antibiotic loaded PMNs [24–26].

In this study, we investigated the *in vitro* effect of MTZ, at doses that are observed clinically in periodontal pockets [27] on PMN-mediated killing of *P. gingivalis*. We found that MTZ modulated PMN activation states to enhance PMN mediated killing of bacteria.

## Materials and methods

### Human subjects

This study was approved by the University of Toronto's Research Ethics Board (30044, 29410). Signed consent was obtained from all participants. Human peripheral blood was drawn from healthy volunteers aged 24 to 30 years old by a trained phlebotomist. Freshly drawn whole blood samples collected into vacutainer containing 0.1 ml volume of sodium citrate as anticoagulant were used to assess the *in vitro* effect of MTZ on PMN responses to *P. gingivalis* according to previously described protocols [28]. We have found from extensive experience that isolating the neutrophils alters their activation state thereby impacting on the degree of activation due to a stimulus [28]. Therefore, whole blood was used to avoid perturbations in PMN activation state and to preserve native PMN surface CD marker expression, which can be altered by standard purification procedures.

### Metronidazole

A stock solution of metronidazole (Habers Pharmacy, Toronto, ON, CA) was prepared in dimethyl sulfoxide (DMSO) at a concentration of 500 μg/ml and stored at −20°C for up to 2 months. Preliminary experiments showed that at the dilutions used, the DMSO added had no effects on the assays described below.

### P. *gingivalis* assay

*P. gingivalis* strain ATCC33277 was grown in Todd-Hewitt broth (Becton Dickinson, MD, USA) supplemented with 5 μg/ml hemin (Sigma-Aldrich, St. Louis, MO, USA) and 1 μg/ml menadione (Sigma Aldrich) (THB-HK) or Blood Agar Base plates (Becton Dickinson) supplemented with 5 μg/mL hemin and 1 μg/mL vitamin K and 5% (v/v) defibrinated sheep blood (Cedarlane, ON, CA). *P. gingivalis* was cultured in an anaerobic chamber (90% N2, 5% CO2 and 5% H2) at 37°C. To test the susceptibility of planktonic *P. gingivalis* to MTZ, bacteria were first grown on agar medium for 72 h then transferred in liquid medium. The minimal inhibitory concentrations (MIC) and minimal eradication concentration (MEC) of planktonic *P. gingivalis* (OD_600_ <0.1) by MTZ were determined by a broth microdilution assay. Briefly, 100 μl of a 10-fold dilution of a 48-h culture of *P. gingivalis* inoculated into fresh THB-HK was mixed with 100 μl of serially diluted MTZ (ranging from 1 ng/ml −2 mg/ml in fresh broth medium) in a 96-well microplate. Control wells with no bacteria or no antibiotic were also prepared. After incubation at 37°C for 48 h under anaerobic conditions the spent medium and free-floating bacteria were removed by aspiration. Bacterial growth was determined visually. To determine the minimum antibiotic concentration at which no viable cell counts were recovered from planktonic culture. In order to test the ability of MTZ to eradicate bacterial cells, aliquots (5 μl) of each well that showed no visible growth were mixed with culture medium in a 96-well microplate and incubated further in the anaerobic incubator at 37°C for 48 h using a modified microdilution assay [29]. The lowest concentration at which no growth was observed was considered the MEC.

For PMN functional studies, MTZ stocks were diluted in Hanks buffer to give a final concentration of 2 μg/ml and 50 μg/ml. MTZ was used at concentrations similar to those achieved in the periodontal pocket during antibiotic therapy [27].

### *P. gingivalis* killing assay

Viability of ATCC33277 was assessed using cells grown on agar medium for 72 h. Bacteria were scraped from the plate and transferred to Hanks balanced salt solution with no magnesium and calcium (Hanks^−/−^ Buffer) at 37°C until they reached (OD600 of 0.4). MOIs of 50 or 150 were calculated for each experiment using a previously generated standard curve. Of note, our group has found from extensive experience that each healthy individual has a slightly different PMN count in blood with an approximate average of 10^5^ cells/ml PMNs into 0.1 ml of human whole blood [28, 30]. One hundred microliters of bacterial culture at an MOI of 50 or 150 was added to 0.1 ml of whole blood to approximate MOI based on the average MOI counts in blood for subsequent experimental incubations. PMNs in whole blood were pretreated for 30 min in the presence of either 2 μg/ml or 50 μg/ml MTZ. Control PMNs were subjected to a similar incubation without antibiotic. To isolate the direct or indirect effect of MTZ on bacterial cells in this experiment, PMNs were washed one time with Hanks^−/−^ Buffer. Pretreated PMN cells (approximately 10^5^ cells/ml) were then incubated with *P. gingivalis* cells at 37°C in an anaerobic chamber for a further 30 min. To determine survival, serially diluted samples were spread on blood agar plates and incubated for 5 days at 37°C in the anaerobic chamber. Surviving colonies were counted to determine % survival relative to untreated controls.

### PMN degranulation/activation assay

PMN stimulation was performed essentially as described previously [30, 31]. Whole blood (approximately 5 x 10^5^) was incubated for 1 h at 37°C with MTZ at different concentrations (2 and 50 μg/ml) and *P. gingivalis* strain at various MOIs (1:50 and 1:150). Pretreated whole blood samples were fixed with 1.6% paraformaldehyde (PFA) for 15 min at 4°C followed by isotonic lysis using BD Pharm Lyse solution. PMNs were resuspended in fluorescent-activated cell sorting buffer (Hanks' balanced salt solution, 1% bovine serum albumin, 2 mM EDTA) and blocked with mouse IgG (2 μg, Sigma) and rat serum (60 to 80 μg, Sigma) for 20 min on ice. A multicolour flow cytometry panel was used to label PMNs for 30 min at 4°C as follows: CD16-AF700 (BioLegend), CD63-PerCP-Cy5.5 (BioLegend), CD66-APC (eBioscience). Appropriate fluorescently tagged isotype control antibodies were used to establish negative staining for each CD marker. Flow cytometer channel voltages were calibrated manually with rainbow beads to normalize sample acquisition on different days. Compensation was performed with single-stained One Comp eBeads (eBioscience). Gating was performed as described previously [21]. At least 2 x 10^4^ gated events were acquired using an LSR Fortessa (BD Biosciences) flow cytometer. Data were analyzed using FlowJo (vX) software. A representative dotplot to demonstrate our gating strategy was included in (Supplementary Figure S1).

### NET and MPO assay

NET formation was assessed by flow cytometric analysis as described by our lab in the past [30]. Briefly, in this flow cytometry-based assay we measured surface expression of citrullinated DNA (a chemically modification of DNA that occurs during NETosis) generated during NETosis on the surface of neutrophils, which are gated using neutrophil specific markers. NETosis was induced by a 3-h incubation of 0.1 ml of whole blood (approximately 5 x 10^5^) with *P. gingivalis* at various MOIs (1:50 and 1:150) in the presence of MTZ (2 or 50 μg/ml). Sample processing and flow cytometry were performed as above. Cells were labeled using an α-MPO-PE (Origene) and a rabbit α-histone H3 (Abcam) primary antibody followed by a α-rabbit-AF488 (Abcam) secondary.

### Phagocytosis assay

To measure phagocytosis, bacterial cells were labeled with pHrodo Red succinimidyl ester (pHrodo; Life Sciences) as described previously [28]. *P. gingivalis* at various MOIs (1:50, and 1:150) was pre-exposed to MTZ (2 or 50 μg/ml) and after incubation the supernatant was removed and 50 μl of 100 mM sodium bicarbonate (pH 8.5) was added. pHrodo Red was then added to the strain at a concentration of 0.5 mM, and incubated at room temperature for 60 min with protection from light. Labeled bacteria were then washed with PBS to remove free dye. Human whole blood was incubated with the labeled *P. gingivalis* for 30 min at 30°C, and processed for flow cytometric analysis as above.

### ROS assay

To measure ROS generation, we used a reduction of cytochrome C assay as described previously [32]. Whole blood samples incubated with *P. gingivalis* strain at various MOIs (1:50 and 1:150) in the presence or absence of MTZ (2 or 50 μg/ml) along with control samples stimulated with phorbol 12-myristate acetate (PMA) were analyzed for respiratory burst activity by cytochrome C reduction. Absorbance of reduced cytochrome C was measured at 550 nm.

### Statistical analysis

One-way ANOVA with the *post hoc* Tukey's test and Student's t test were used to compare neutrophil activation by experimental groups. *P* ≤ 0.05 was considered statistically significant. Data were analyzed with IBM SPSS Statistics (version 24) statistical software.

## Results

### MTZ potentiates PMN mediated killing of *P. gingivalis*

**Figure 1:**
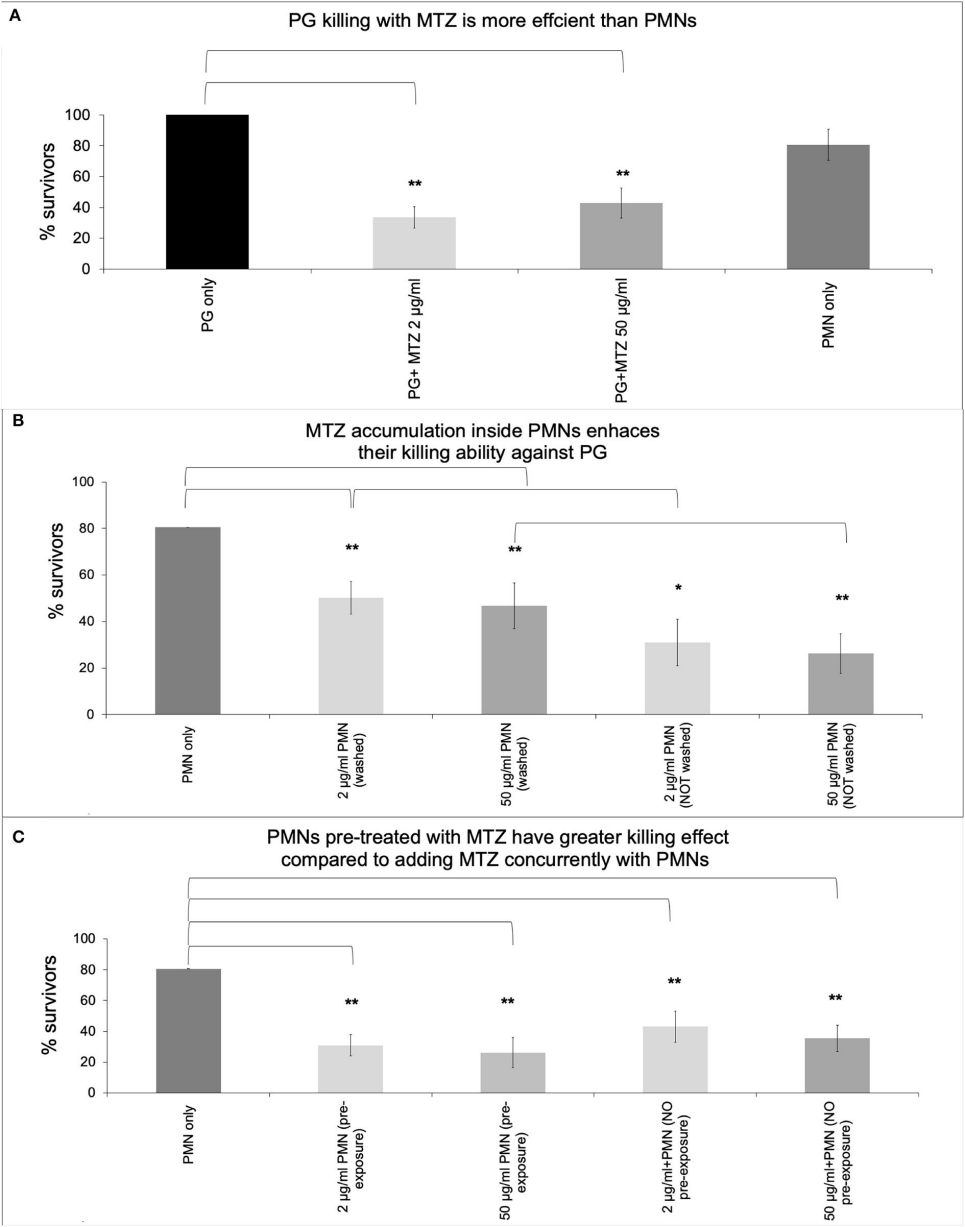
MTZ promotes *P. gingivalis* killing by PMNs. *P. gingivalis* was added to tubes containing **(A)** MTZ (2 or 50 μg/ml) or PMNs alone, **(B)** PMNs that had been pre-loaded with MTZ (30 min treatment) and then washed or not washed and added to *P. gingivalis*, or **(C)** PMNs immediately following addition of MTZ. All tubes were incubated at 37°C for further 30 min and aliquots were removed, washed to remove antibiotics and diluted for assessment of bacterial killing. PMNs and MTZ were added together with an MOI of 150 bacteria per PMN. Results represent mean CFU counts ± standard deviation. Statistically significant differences are indicated (^**^P ≤ 0.05 and ^*^P ≤ 0.1, Student's t test, ANOVA). Mean % survivors ± SEM are indicated from four independent repeats.

In order to determine whether MTZ alters PMN mediated killing of *P. gingivalis*, we examined PMN killing ability in the presence and absence of antibiotic. Firstly, the antibacterial activity of MTZ was assessed against *P. gingivalis* by a broth microdilution assay to confirm that the MIC is comparable to those found in other studies (Table 1). The MIC for MTZ was 2 μg/ml which fell within the range that has been previously reported (0.02 to 2.1 μg/ml) [27, 33–36]. Next, periodontal pathogens were incubated with either 2 μg/ml or 50 μg/ml MTZ or human PMNs in whole blood to assess if they were more susceptible to killing by antibiotic than normal PMNs. We found that MTZ was more effective than human PMNs at killing *P. gingivalis* (surviving CFU of 33 % with 2 μg/ml MTZ and 42% with 50 μg/ml MTZ treatment vs. 80% with human PMNs) (Figure 1A). To determine if MTZ can potentiate PMN mediated killing of *P. gingivalis*, PMNs and MTZ were added together with an MOI of 150 bacteria per PMN. PMNs were pretreated with MTZ (2 μg/ml or 50 μg/ml) for 30 min before adding to the bacteria, and this produced more effective bacterial killing of *P. gingivalis* than either MTZ alone or untreated PMNs (*P* <0.05) (Figure 1B). When the PMNs were maintained with MTZ after pretreatment, the combined killing effect was even greater than when the MTZ was washed out. When MTZ and PMN were added without a pre-treatment the effect on killing of *P. gingivalis* was essentially the same as MTZ alone. The more pronounced effect in killing *P. gingivalis* was observed with MTZ-loaded PMN suggesting that metronidazole synergize with neutrophil function in the process of eradicating *P. gingivalis* (Figure 1C). While it remains unclear for now if MTZ accumulated inside PMN, further studies are needed along this line.

**Table 1:** Minimal inhibitory concentration (MIC) and minimal eradication concentration (MEC) value of metronidazole against ATCC 33277 strain of *P. gingivalis*.

	**Metronidazole**	
Strain	MIC	MEC
ATCC 33277	2 μg/ml	2 μg/ml

### MTZ enhances PMN degranulation

**Figure 2:**
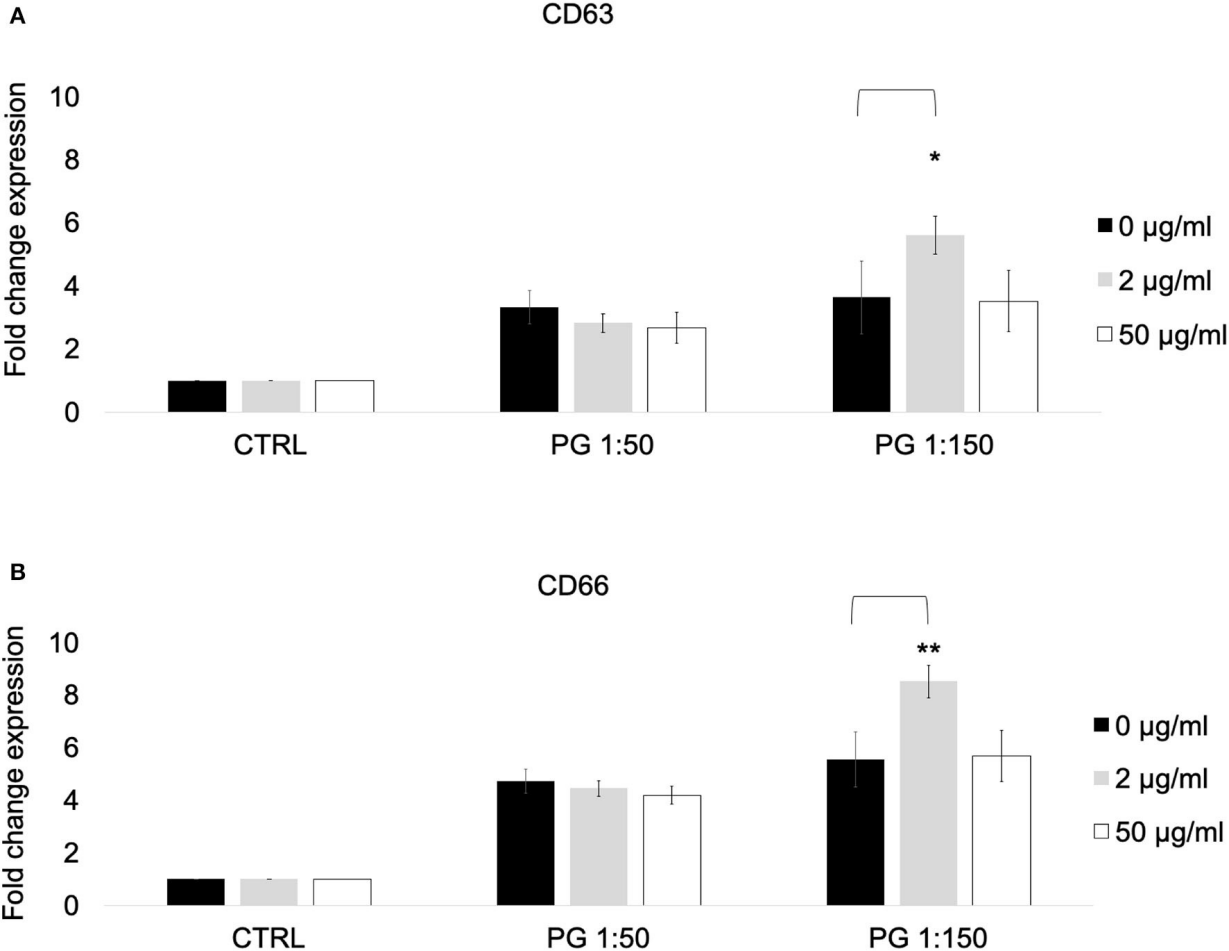
MTZ increased the expression of PMN markers of degranulation and activation. PMNs were incubated at 37°C for 30 min in the presence of MTZ as indicated. Cell surface CD markers of **(A)** degranulation and **(B)** activation were analyzed by flow cytometry. Fold change in expression ± SEM are shown for 4 independent repeats. Analysis of variance with one-way ANOVA followed by a Student's t test was performed to determine statistical significance. ** *P* ≤ 0.05 and *P ≤ 0.1.

Since our results revealed that treatment with MTZ can potentiate PMN mediated killing of *P. gingivalis*, we tested the effects of MTZ on various PMN functions. Whole blood was used for our flow cytometry assay to minimize the absolute increase in activation that would occur in cells due to their pre-activation during isolation. To ensure that our CD marker assay results were specific to neutrophils, we restricted our data acquisition to granulocytes using the SSC area (SSC-A) by FSC area (FSC-A) and then gated on neutrophils by using CD16 and CD66 in our gating strategy. To confirm this, the cells were sorted using a BD FACSAria cell sorter on medium pressure, and it was confirmed by microscopy that only neutrophils were selected. We first assessed the effects of MTZ on PMN expression of specific cell surface CD makers associated with PMN activation and degranulation. We found that the surface expression of CD66 and CD16 in the presence of MTZ at concentrations of 2 and 50 μg/ml was not significant different from the unstimulated controls (absence of MTZ) suggesting that MTZ alone had no effect on PMN activation. When MTZ treated PMNs were exposed to *P. gingivalis* at an MOI of 1:150, we found that PMN expression of CD66 and CD63 were increased relative to PMNs that were not exposed to the low dose of MTZ (2 μg/ml). This effect was not apparent at the high concentration of MTZ (50 μg/ml) (Figures 2A–B). A representative histogram for this experimental condition in our cytometry experiments is shown in Supplementary Figure S2.

### MTZ did not inhibit NETosis

**Figure 3:**
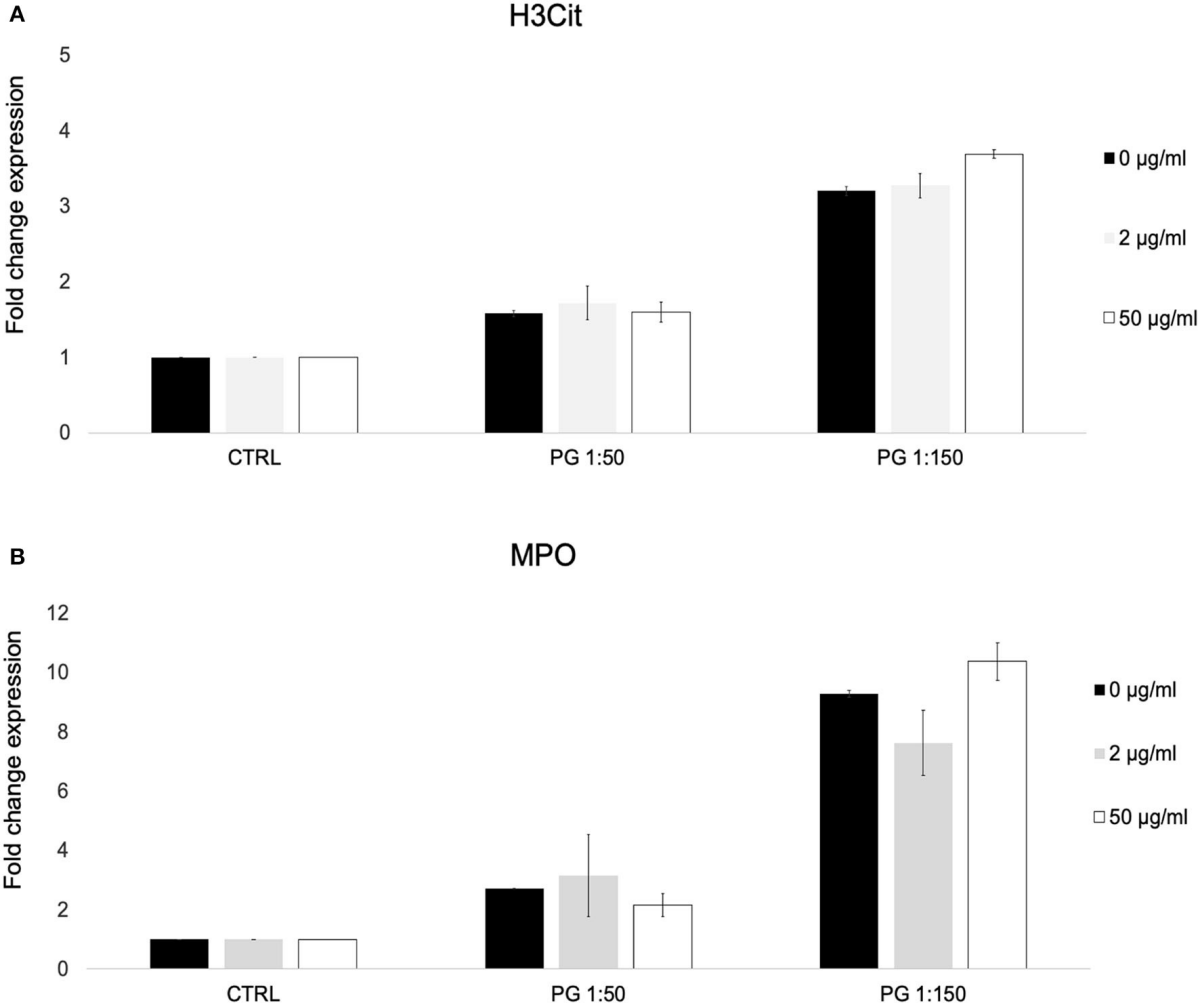
MTZ did not significantly alter PMN surface expression of markers of neutrophil extracellular trap formation. PMNs were incubated at 37°C for 30 min in the presence of MTZ as indicated. The cells were labeled with **(A)** H3Cit and **(B)** myeloperoxidase (MPO) antibodies and analyzed by flow cytometry. Bar graphs show fold change expression of each marker ± SEM. Mean values from four independent experiments are shown.

We used a previously described flow cytometric assay for NETosis to identify citrulinated histone H3 (H3Cit) and myeloperoxidase (MPO) on the cell surface of PMNs exposed to *P. gingivalis* in the presence or absence of MTZ. We found that MTZ did not exert an effect on NET formation by *P. gingivalis* stimulated PMNs. While exposure of PMNs to *P. gingivalis* alone greatly induced H3Cit and MPO expression by human blood PMNs, which was proportional to the MOI, MTZ did not induce a significant further increase in NETosis. The high concentration of MTZ (50 μg/ml) did lead to a small increase of expression of the NETosis markers, but this was not statistically significant (Figures 3A–B). A representative histogram for this experimental condition in our cytometry experiments is shown in Supplementary Figure S3.

### MTZ suppresses PMN phagocytosis of *P. gingivalis*

**Figure 4:**
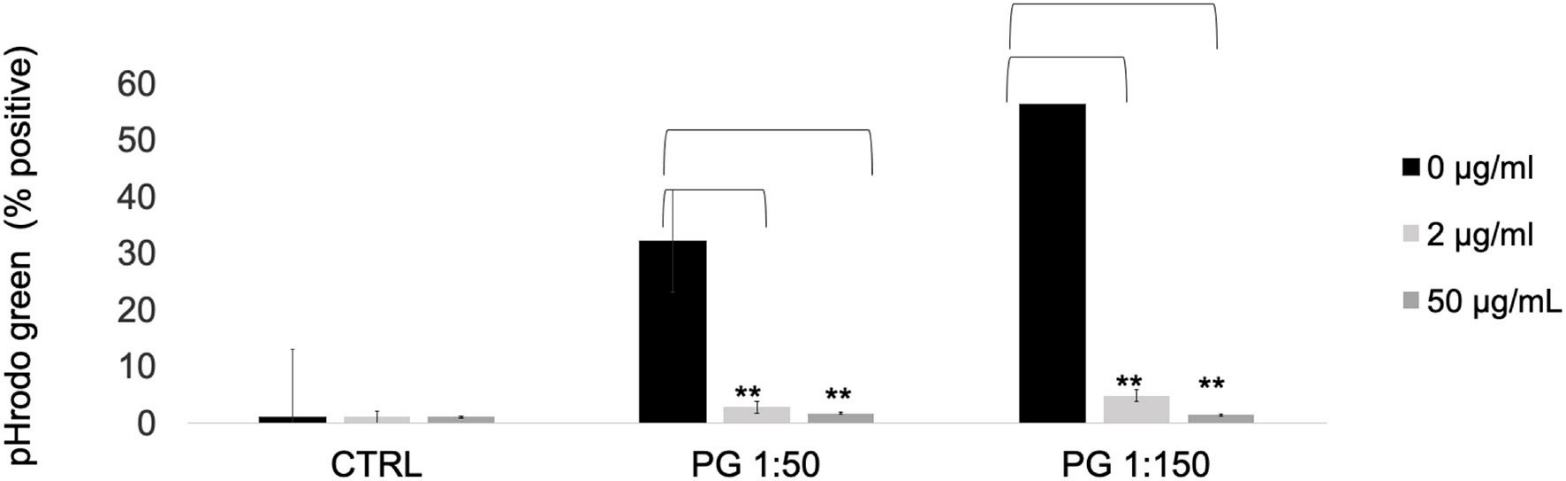
MTZ inhibits PMN phagocytosis. *P. gingivalis* was labeled with pHrodo Red for 1 h. PMNs with or without MTZ treatment were then incubated with labeled bacteria for 1 h at 37°C. The percentage of pHrodo positive cells was determined by flow cytometry. Statistically significant differences are indicated (**P ≤ 0.05, Student's t test). Mean values ± SEM from five independent repeats are shown.

To determine the effects of MTZ on the phagocytic capacity of PMNs, we incubated pHrodo-labeled *P. gingivalis* bacteria with MTZ treated or untreated PMNs for 1 hr at 37°C. At both the high and the low concentrations of MTZ, pHrodo positive PMNs were greatly reduced, indicating a strong anti-phagocytosis effect of MTZ (Figure 4).

### MTZ suppresses PMN ROS production in response to *P. gingivalis*

**Figure 5:**
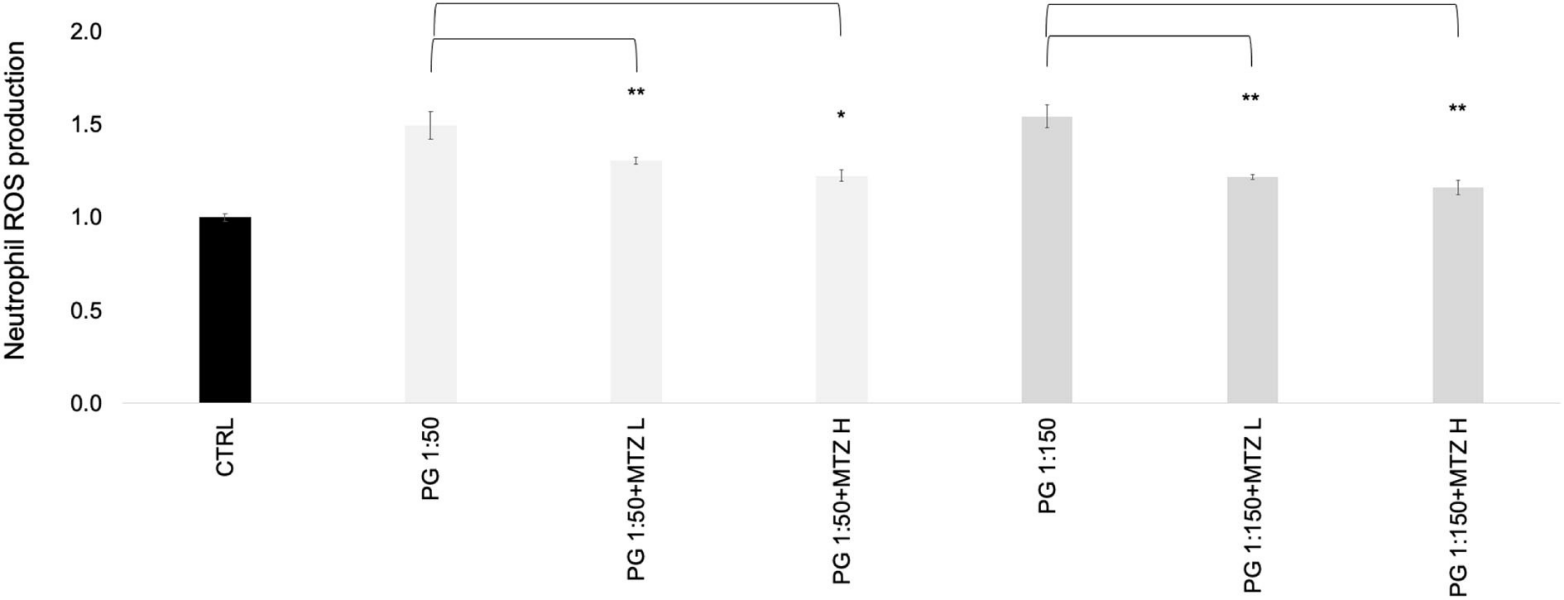
MTZ inhibits PMN ROS production. Respiratory burst activity was determined in PMN stimulated with *P. gingivalis* in the presence or absence of MTZ. Reduction of cytochrome C was measured by spectrophotometry in MTZ treated cells relative to that for controls. Statistically significant differences are indicated (**P ≤ 0.05, *P* ≤ 0.1 Student's t test). Mean values ± SEM from five independent experiments are shown.

We investigated the effects of MTZ on ROS production by human blood PMNs by cytochrome C assay. We found that *P. gingivalis* alone induced PMN ROS production, however when MTZ was added it suppressed this effect (Figure 5).

## Discussion

Conventional periodontal treatment involving scaling and root planning procedures is highly effective at resolving mild cases of periodontitis, however satisfactory clinical outcomes in periodontitis patients often cannot be achieved. In such cases, adjunctive therapies may be appropriate, more specifically the use of highly effective antibiotics. MTZ is a front-line choice for treatment of *P. gingivalis* mediated periodontal disease [37, 38]. Antibiotics including MTZ have been previously reported to have immunomodulatory effects to potentiate the properties of different immune cell types, including PMNs [39, 40]. The current study provides evidence that MTZ, at concentrations attainable *in vivo*, can potentiate PMN mediated killing of *P. gingivalis*, in addition to its direct antimicrobial activities, which could contribute to its therapeutic mechanism of action in treatment of periodontal disease. When PMNs in whole blood were incubated with MTZ they killed significantly more bacteria than either MTZ alone or untreated PMNs. The lower concentration of MTZ used in this study was as effective as the higher concentration with respect to potentiation of PMN killing activity, and both concentrations are within the normal therapeutic range typically found in gingival crevicular fluid (0.2 to 50 μg/ml) [27]. Our experiments were performed with whole blood therefore it is possible that other cells could contribute to the killing of the bacteria phenotype. Although, neutrophils normally make up 70–90% of the immune cells present other cells could be impacting the elimination of the bacteria as a response to the antibiotic treatment.

We used whole blood as PMNs are much more stable in whole blood compared to isolated PMNs, where we would expect a lot of cell death. PMNs entering the deep pockets of the periodontal lesions are known to eliminate pathogens by oxygen-dependent and oxygen-independent means. Importantly, these processes can occur in hypoxic periodontal pockets where oxygen concentration is as low as 1–3% [41]. While cell death can occur under anaerobic conditions, it doesn't seem to prevent PMNs ability to kill anaerobic pathogens in the presence or absence of oxygen [42]. This is consistent with our results obtained in the anaerobic chamber experiments for 30 min where neutrophil viability was not altered (unpublished data).

It is feasible that adjunctive use of MTZ can enhance the PMN killing of *P. gingivalis* in patients with periodontitis. MTZ is able to accumulate in the tissue, including white blood cells at the site of infection. However, a receptor for MTZ uptake has not been described yet and it is unclear whether it is transported by an active or passive process [43].

In addition to examining the killing capability of MTZ—pre-exposed PMNs against *P. gingivalis*, we investigated the effect of MTZ on the functional changes of PMNs in whole blood by flow cytometry. We found that low dose (2 μg/ml) MTZ promoted PMN degranulation, as reflected by increased surface expression of two granule markers (CD66 and CD63). However, we did not observe the same effect at the higher dose of MTZ (50 μg/ml), which was nonetheless effective at enhancing PMN mediated bacterial killing. We recognize that blood factors (other cells and serum components) impact on the activation of the neutrophils using our whole blood approach but we believe that isolating neutrophils and stimulating exogenously would have less clinical significance than assessing the effect of metronidazole on neutrophils in blood. Therefore, there are also tradeoffs to using isolated PMNs as well and opted for the more clinically relevant blood approach. This is relevant as oral sourced bacteremia is a real clinical concern and identifying the possibly effectiveness of an antibiotic on neutrophil mediated killing of bacteria in blood is clinically important. Although we cannot say with certainty that other immune cells did not also contribute to the bacterial killing phenotype, it is certain, based on PMN surface markers, that the PMNs are becoming activated and therefore it seems unlikely that PMNs would not have a role in the killing mechanism, possibly in conjunction with other blood factors.

Furthermore, we found that MTZ suppressed *P. gingivalis* induced phagocytosis and ROS production, and had no effect on NETosis. Together this suggests that MTZ might not directly promote PMN-mediated bacterial killing, but rather that the combined effects of normal PMN killing mechanisms, such as degranulation and NETosis, might combine to produce an especially lethal milieu for the bacteria. We acknowledge that other cells besides neutrophils could contribute to NETs. In our flow cytometry-based assay we measured surface expression of NETs on the surface of neutrophils, which were gated using neutrophil specific markers as our lab used this in the past [30]. However, it is still possible that other cells are contributing NETs or contributing to the overall inflammatory state of neutrophils.

The observation that MTZ suppresses rather than promotes two important killing mechanisms of PMNs, phagocytosis and ROS, underscores this interpretation of our results, and suggests that degranulation and/or NETosis must be contributing strongly to the synergistic killing of *P. gingivalis* that we observed with MTZ loaded PMNs. One possibility is that MTZ interferes with P. gingivalis' resistance against granule-derived antimicrobial agents [10]. Future experiments are necessary to further elucidate the mechanism of MTZ-PMN synergistic bacterial killing.

Collectively, we provide evidence that MTZ may function in concert with oral PMNs, which occur constitutively in the gingival crevice, and this may account for its efficacy as a therapeutic option for the treatment of invasive *P. gingivalis* infections including those refractory to conventional treatment.

## Data availability statement

The original contributions presented in the study are included in the article/Supplementary material, further inquiries can be directed to the corresponding author.

## Ethics statement

The studies involving human participants were reviewed and approved by the University of Toronto's Research Ethics Board (30044, 29410). Written informed consent was obtained from all participants for their participation in this study.

## Author contributions

Conceptualization: MG, AB, and MAS. Methodology: MG and MAS. Investigation: MAS, MO, and CS. Formal analysis and writing-original draft preparation: MAS. Review and editing: NF, MG, and MAS. Supervision and project administration: MG. All authors have read and agreed to the published version of the manuscript.

## Funding

This study was conducted with support from Mitacs Accelerate Program (IT07299) as industrial postdoctoral fellowship toward MAS. The authors greatly acknowledge the industrial partner OraVital Inc. for providing financial support for the study. The funders had no role in study design, data collection and analysis, decision to publish or preparation of the manuscript.

## Conflict of interest

Author AB was employed by OralVital Inc. The remaining authors declare that the research was conducted in the absence of any commercial or financial relationships that could be construed as a potential conflict of interest.

## Publisher's note

All claims expressed in this article are solely those of the authors and do not necessarily represent those of their affiliated organizations, or those of the publisher, the editors and the reviewers. Any product that may be evaluated in this article, or claim that may be made by its manufacturer, is not guaranteed or endorsed by the publisher.
