# Supplementary material for: Metronidazole enhances killing of Porphyromonas gingivalis by human PMNs
Source: Front Oral Health. 2022 Aug 29;3:933997. doi: 10.3389/froh.2022.933997 (PMC9464935; doi:10.3389/froh.2022.933997)
Supplement: Supplementary file 1 [file Image_1.pdf]

Supplementary Figure 1

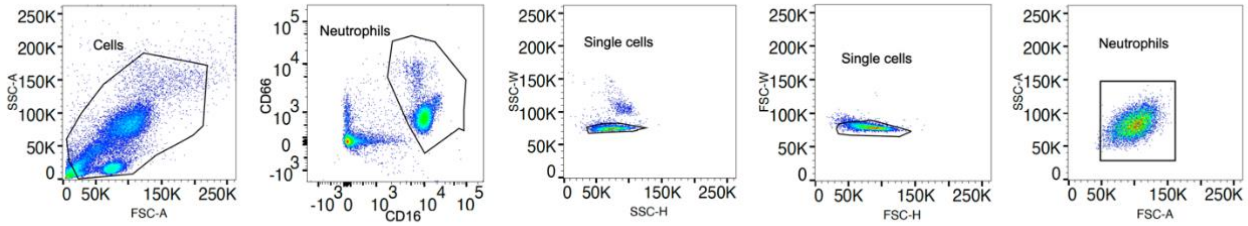

Supplementary Figure S1. Gating strategy for blood neutrophils. Representative blood neutrophil samples from healthy individuals are shown to demonstrate the flow cytometry gating strategy used. Neutrophils were gated in whole blood using CD16hi and CD66hi. Doublets were excluded based on SSC-W x SSC-W X FSC-H. At least $2x10^4$ gated events were acquired per sample.
