# Supplementary material for: Metronidazole enhances killing of Porphyromonas gingivalis by human PMNs
Source: Front Oral Health. 2022 Aug 29;3:933997. doi: 10.3389/froh.2022.933997 (PMC9464935; doi:10.3389/froh.2022.933997)
Supplement: Supplementary file 3 [file Image_3.pdf]

Supplementary Figure S3

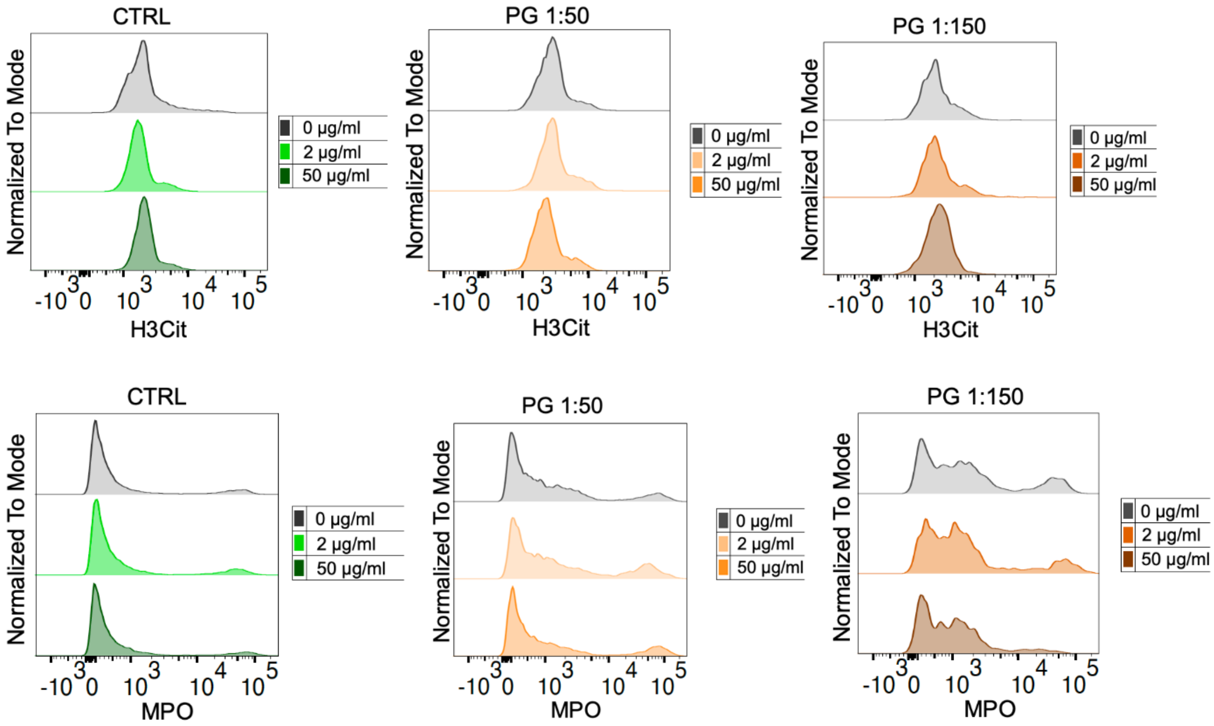

Supplementary Figure S3. Representative histogram of H3Cit and MPO expression demonstrating that MTZ does not activate the expression of specific markers of NETosis. CTRL (green), PG 1:50 (orange), and PG 1:150 (brown) are shown.
